# GSPE reduces lead-induced oxidative stress by activating the Nrf2 pathway and suppressing miR153 and GSK-3β in rat kidney

**DOI:** 10.18632/oncotarget.15033

**Published:** 2017-02-02

**Authors:** Biying Liu, Haili Zhang, Xiao Tan, Daqian Yang, Zhanjun Lv, Huijie Jiang, Jingjing Lu, Ruiqi Baiyun, Zhigang Zhang

**Affiliations:** ^1^ College of Veterinary Medicine, Northeast Agricultural University, Harbin 150030, China

**Keywords:** grape seed procyanidin extract, kidney injury, oxidative stress, Nrf2, nephroprotective effect

## Abstract

Lead (Pb) is a global environmental health hazard that leads to nephrotoxicity. However, the effective treatment of Pb-induced nephrotoxicity remains elusive. Grape seed procyanidin extract (GSPE) has beneficial properties for multiple biological functions. Therefore, the present study investigated whether GSPE reduced Pb-induced nephrotoxicity as well as the protective mechanism of GSPE in a well-established 35-day Pb induced nephrotoxicity rat model. The results showed that GSPE normalized Pb-induced oxidative stress, histological damage, inflammatory, apoptosis, and changes of miR153 and glycogen synthase kinase 3β (GSK-3β) levels in rat kidney. Moreover, GSPE enhanced the induction of phase II detoxifying enzymes (heme oxygenase-1 and NAD(P)H quinone oxidoreductase 1) by increasing nuclear factor-erythroid-2-related factor 2 (Nrf2) expression. This study identifies for the first time that Pb-induced oxidative stress in rat kidney is attenuated by GSPE treatment via activating Nrf2 signaling pathway and suppressing miR153 and GSK-3β. Nrf2 signaling provides a new therapeutic target for renal injury induced by Pb, and GSPE could be a potential natural agent to protect against Pb-induced nephrotoxicity.

## INTRODUCTION

Lead (Pb) is a global environmental health hazard that poses a substantial risk to humans and animals. Pb exposure affects the nervous, immune, renal, skeletal, and hematopoietic systems [1]. Furthermore, Pb easily penetrates through biological membranes and exhibits accumulation patterns in tissues [2]. Kidney is one of the main excretion pathways of Pb in organisms and is a target organ for Pb cytotoxicity [3]. Pb is often released in the environment from natural and anthropogenic sources. Therefore, nephrotoxicity of Pb is a significant public health concern.

At present, the mechanism(s) underlying Pb-induced nephrotoxicity development remain poorly understood. Evidence from animal studies suggests an important factor of lipid peroxidation and degradation of phospholipids in kidney damage is caused by Pb toxicity, which leads to a loss of membrane integrity [4]. Besides, when cells are overwhelmed by oxidative stress, genes involved in cell death signaling are activated to induce apoptosis or necrosis to remove irreversibly damaged cells [5]. Therefore, an increase in the level of apoptosis may underline the pathogenesis of kidney injury. Furthermore, oxidative stress and inflammation, which are tightly linked, are characteristic features of kidney disease [6]. Nuclear factor-κB (NF-κB) activity is inducible in all cell types and regulates many genes involved in inflammatory responses [7]. NF-κB can be stimulated by Pb and is a major factor in pathological conditions of the kidney [8]. Thus, attenuated oxidative stress may serve as a potential mechanism for renal toxic injury and disease induced by Pb.

Glycogen synthase kinase 3β (GSK-3β) is a redox sensitive signaling molecule that plays a pivotal role in a multitude of signaling pathways, including NF-κB, nuclear factor-erythroid-2-related factor 2 (Nrf2), and others. Remarkably, Nrf2 activates antioxidant enzymes and inhibits oxidative stress induced damage [9]. The self-protective antioxidant effects of Nrf2 are controlled by GSK-3β, which promotes the nuclear export and degradation of Nrf2 upon oxidative stress, culminating in switching off the Nrf2 antioxidant response [10]. The switching on and off of Nrf2 protects cells against free radical damage, apoptosis, and promotes cell survival [11]. Interestingly, a recent study showed that the enforced expression of miR153 elicited a post-transcriptional repression of Nrf2 [12]. However, it is still unclear whether Nrf2 plays a role in nephrotoxicity of Pb.

Currently, Pb poisoning is commonly treated by chelating agents such as disodium edetate calcium and sodium dimercaptosuccinate in clinics. Pb forms an insoluble complex with chelating agents to allow its removal from the Pb-burdened tissue, but these chelating agents are incapable of removing metal from intracellular sites and may cause a redistribution of the toxic metal, essential metal loss, and liver or renal dysfunction [13]. Therefore, it is important to develop safe and effective treatments for Pb poisoning.

Grape seed procyanidin extract (GSPE) belongs to a larger group of polyphenolic compounds and contains oligomers and polymers of monomeric flavonoids [14]. In addition to antioxidant, GSPE has antiviral, antibacterial, anti-inflammatory, and anti-apoptotic effects [15, 16]. Remarkably, GSPE also has unique properties that prevent kidney injury induced by colistin [17], cisplatin [18], and cyclosporine A [19]. The specific role of GSPE in Pb-induced nephrotoxicity is unknown.

We hypothesized that GSPE could suppress nephrotoxicity of Pb. To investigate this, we measured the regulatory effects of GSPE on the Nrf2 signaling pathway induced by Pb and the mechanisms involved in the GSPE-mediated protection against Pb-induced nephrotoxicity.

## RESULTS

### GSPE attenuates Pb-induced renal damage

**Figure 1:**
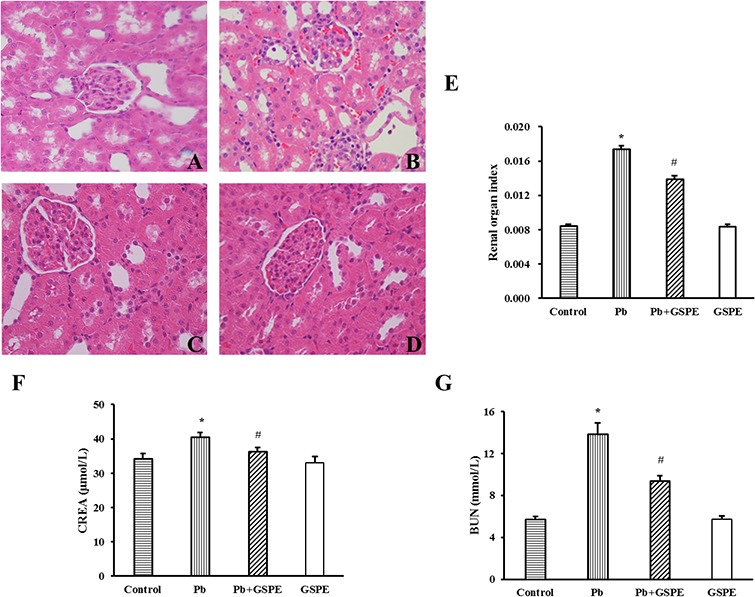
The protective effect of GSPE on Pb-induced renal damage Histopathological changes in renal tissues: Paraffin sections of kidney tissues from **A**. control, **B**. Pb-treated, **C**. Pb + GSPE, and **D**. GSPE were stained with hematoxylin and eosin (×400). **E**. The organ index of the kidney in rats. **F**. CREA level of kidney in rats. **G**. BUN level of kidney in rats. Data are expressed as mean ± SEM, n = 10. * Significantly different (P < 0.05) from control group, and ^#^ significantly different (P < 0.05) from Pb-treated group.

Histopathological change is a direct indication of renal injury. Hematoxylin and eosin stained renal tissues showed normal kidney tubules and corpuscles in the control group (Figure 1A) and GSPE group samples (Figure 1D). In contrast, Pb-induced histopathological changes were observed in the renal tissues including the destruction of tubular structures, necrosis and disorganization, inflammatory cell infiltration, vacuolar degeneration, and hyperemia of the renal interstitium (Figure 1B). However, coadministration of GSPE significantly diminished Pb-induced epithelial atrophy, necrosis, and hyperemia (Figure 1C). We measured the renal-organ index (Figure 1E), and GSPE significantly (P < 0.05) restored kidney weight when compared with rats treated with Pb alone. Results in the GSPE alone group did not differ significantly from the normal control group.

Creatinine (CREA) (Figure 1F) and blood urea nitrogen (BUN) (Figure 1G) levels were significantly higher in the Pb-treated group compared with the control group (P < 0.05). GSPE alone did not increase CREA and BUN levels, but significantly (P < 0.05) inhibited their increase induced by Pb.

### GSPE ameliorates Pb-induced oxidative stress in the kidney

**Figure 2:**
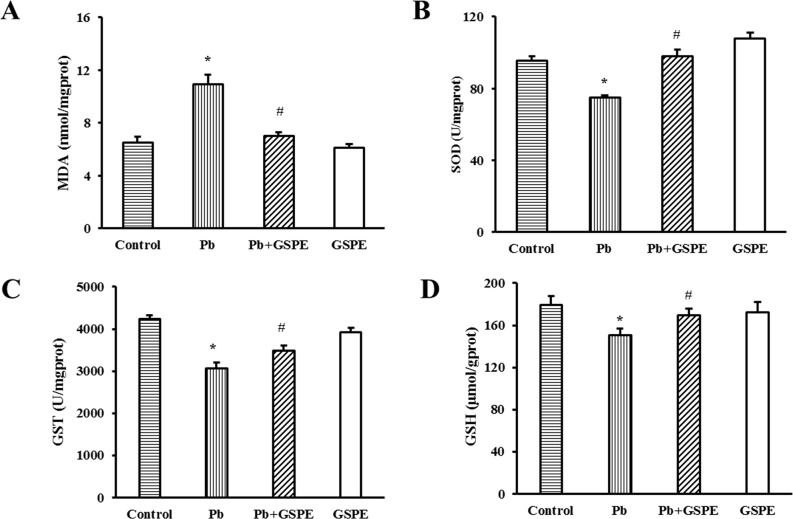
Effects of GSPE on oxidative stress indicators in Pb-treated rat kidneys Samples were collected from control, Pb-treated group, Pb + GSPE group, and GSPE. **A**. The concentration of MDA in the kidney. **B**. The activity of SOD in the kidney. **C**. The activity of GST in the kidney. **D**. The concentration of GSH in the kidney. Data are expressed as mean ± SEM, n = 10. * Significantly different (P < 0.05) from control group, and ^#^ significantly different (P < 0.05) from Pb-treated group.

We initially investigated specific indicators of oxidative stress, including the concentration of malondialdehyde (MDA) and glutathione (GSH), and the activity of superoxide dismutase (SOD) and glutathione S-transferase (GST). As expected, Pb administration for 5 weeks induced a significant increase in kidney MDA level compared with the control rats (Figure 2A, P < 0.05). The coadministration of GSPE markedly attenuated the lipid peroxidation. The activities of SOD (Figure 2B) and GST (Figure 2C) in the kidney were significantly decreased in the Pb-treated group (P < 0.05), and this effect was reversed significantly by GSPE treatment. In addition, we found that Pb treatment significantly reduced GSH level, however, GSPE prevented the decrease of GSH caused by Pb (Figure 2D, P < 0.05).

### GSPE decreases total Pb concentrations in the kidney

**Figure 3:**
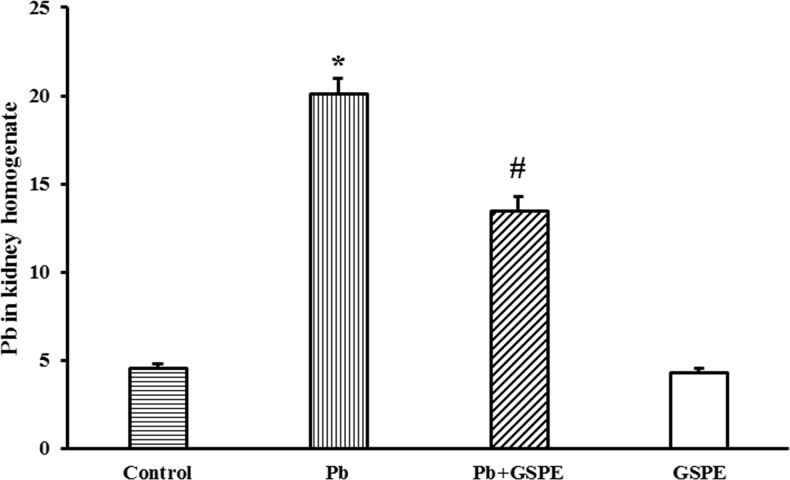
Total Pb accumulation in rat kidney Data are expressed as mean ± SEM, n = 10. * Significantly different (P < 0.05) from control group, and ^#^ significantly different (P < 0.05) from Pb-treated group.

To examine how GSPE prevents Pb-induced renal toxicity, Pb retention in the kidney was measured (Figure 3). The total concentration of Pb in the Pb-treated group was significantly higher than the control group (P < 0.05). GSPE significantly attenuated Pb retention in the kidney (P < 0.05).

### GSPE relieves renal inflammation

**Figure 4:**
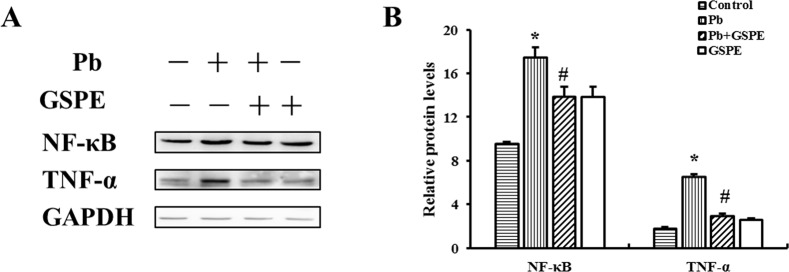
GSPE inhibited Pb-induced pro-inflammatory signals: the levels of NF-κB and TNF-α in the kidney **A**. The protein levels of NF-κB and TNF-α. **B**. The values from densitometry of NF-κB and TNF-α were normalized to the level of GAPDH protein and expressed as fold increased. Data are expressed as mean ± SEM of 4 independent experiments. *Significantly different (P < 0.05) from control group, and ^#^significantly different (P < 0.05) from Pb-treated group.

As shown in Figure 4A, NF-κB and tumor necrosis factor-α (TNF-α) protein levels were increased in the Pb treatment group. However, GSPE efficiently decreased Pb-induced NF-κB and TNF-α levels.

### GSPE ameliorates Pb-induced nephrocyte apoptosis

**Figure 5:**
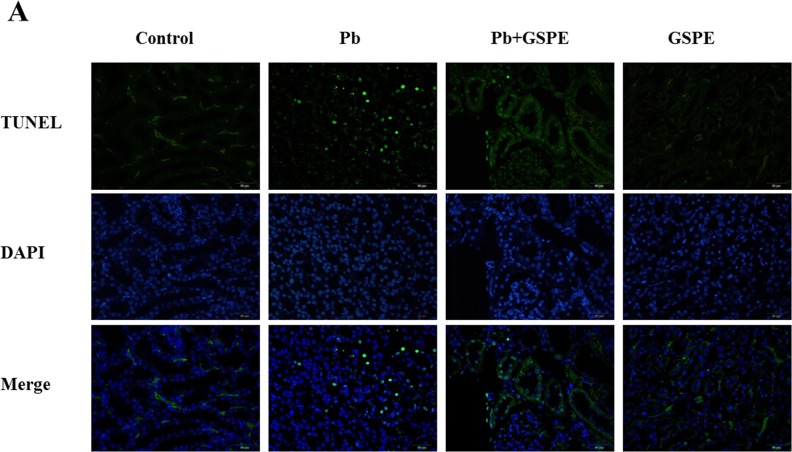

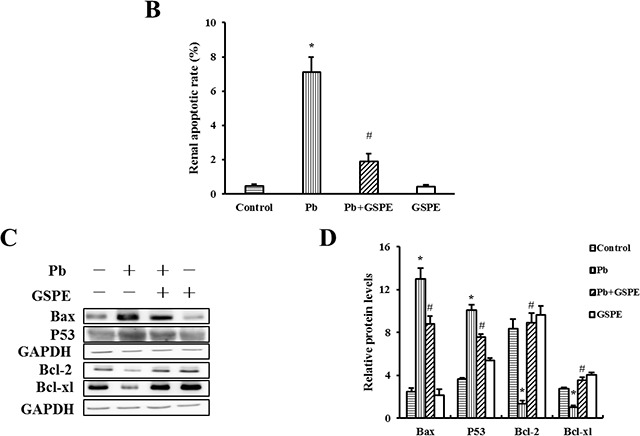
GSPE ameliorates Pb-induced nephrocyte apoptosis **A**. Apoptosis was detected by in situ TUNEL (green) and DAPI (blue). **B**. Quantitative analysis of TUNEL-positive cells content. **C**. The protein levels of Bax, P53, Bcl-2, and Bcl-xl. **D**. The values from densitometry of Bax, P53, Bcl-2, and Bcl-xl were normalized to the level of GAPDH protein and expressed as fold increased. Data are expressed as mean ± SEM of 4 independent experiments. * Significantly different (P < 0.05) from control group, and ^#^ significantly different (P < 0.05) from Pb-treated group.

TUNEL staining was used to assess apoptosis in the kidney. As shown in Figure 5A and 5B, Pb exposure induced an obvious increase in TUNEL positive cells in renal areas compared with the control group. GSPE treatment with Pb exposure strongly decreased the rate of Pb-induced renal cell death in the kidney.

To further establish the protective role of GSPE in Pb-induced nephrocyte apoptosis, we measured the related apoptotic proteins Bax, P53, Bcl-2, and Bcl-xl. Our results showed that Pb increased the levels of Bax, P53, and decreased the levels of Bcl-2 and Bcl-xl (Figure 5C and 5D). Notably, apoptosis in the kidney was inhibited by GSPE.

### GSPE inhibits the activation of GSK-3β induced by Pb

**Figure 6:**
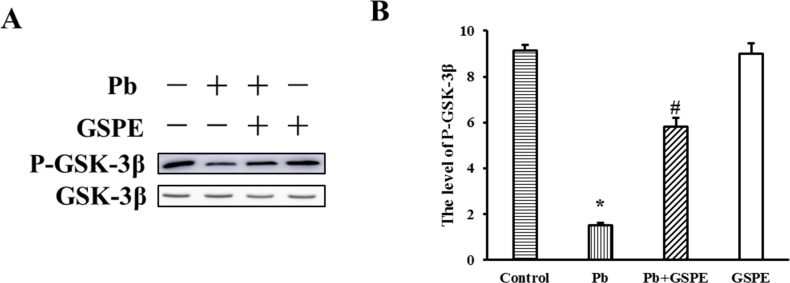
Effect of GSPE on the level of P-GSK-3β in Pb-induced nephrotoxicity **A**. The protein levels of P-GSK-3β and GSK-3β. **B**. The values from densitometry of P-GSK-3β were normalized to the level of GSK-3β protein and expressed as fold increased. Data are expressed as mean ± SEM of 4 independent experiments. * Significantly different (P < 0.05) from control group, and ^#^significantly different (P < 0.05) from Pb-treated group.

GSK-3β is situated at the nexus of numerous signaling pathways. GSK-3β is inactivated on phosphorylation at Ser9. Therefore, we determined the phosphorylation status of GSK-3β at Ser9 residue by western blot analysis. Our results showed that Pb decreased the level of P-GSK-3β (Figure 6), and this was inhibited by GSPE.

### GSPE activates the Nrf2 pathway in Pb-treated rat

**Figure 7:**
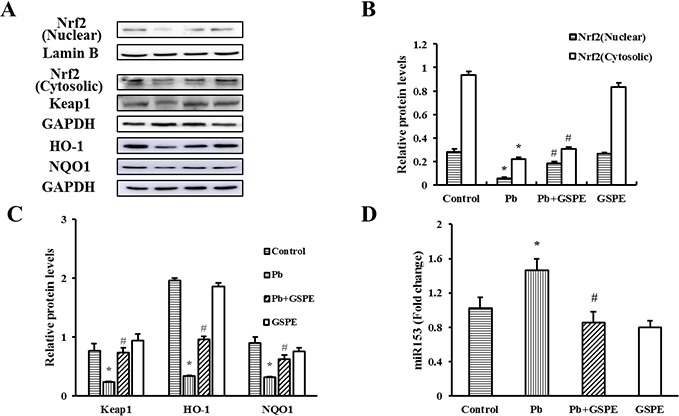
Effects of GSPE on the levels of Nrf2, Keap1, NQO1, HO-1 and miR153 in Pb-treated kidney **A**. The protein levels of Nrf2 (nuclear and cytosolic), Keap1, NQO1, and HO-1. **B**. The values from densitometry of Nrf2 (nuclear) are normalized to the level of Lamin B protein and expressed as fold increased. **B-C**. The values from densitometry of Nrf2 (cytosolic), Keap1, NQO1, and HO-1 are normalized to the level of GAPDH protein and expressed as fold increased. **D**. Quantification of the level of miR153. Data are expressed as mean ± SEM of 4 independent experiments. miRNA expression is shown as fold change with respect to control group. * Significantly different (P < 0.05) from control group, and ^#^ significantly different (P < 0.05) from Pb-treated group.

Nrf2 nuclear translocation is a necessary step of Nrf2 activation. First, we measured the protein levels of Nrf2 (nuclear and cytosolic), Kelch-like-ECH-associated protein 1 (Keap1), and Nrf2-target genes (NAD(P)H quinone oxidoreductase 1 (NQO1) and heme oxygenase-1 (HO-1)) (Figure 7A-7C). Next, relative quantitative real time RT-PCR analysis for miR153 was performed (Figure 7D). We found that Pb treatment decreased the levels of Nrf2, Keap1, NQO1, and HO-1, whereas miR153 expression was increased. However, these effects were reversed by GSPE.

## DISCUSSION

Several studies have reported that Pb is non-biodegradable, persists in the environment, and induces a variety of adverse effects over a long period. The chronic ingestion of Pb causes various well-documented pathologies in the kidney following its intestinal absorption and subsequent accumulation [20].

The serum levels of BUN and CREA can be interpreted as indices of renal dysfunction [21, 22]. Histopathological changes and the renal organ index are indicators used to evaluate the toxicity of Pb in rats [23]. In this study, histopathological changes, CREA and BUN levels, and renal organ index suggested that Pb caused kidney damage, while GSPE reversed it. Therefore, GSPE could be a useful therapeutic drug to inhibit the progression of Pb-induced nephrotoxicity.

The excretion of Pb in the urine reflects a high concentration of Pb in the posterior kidney. Our results revealed that GSPE attenuated the specific bioaccumulation and retention of Pb in the kidney. Based on previous studies, Pb is incapable of inducing the production of metallothionein [24] and being a divalent cation, it is unlikely to be a permanent species. However, Pb has a negative impact on membrane structure and functionality, including lipid oxidation and membrane fluidity [25], which are partially responsible for Pb accumulation. Our studies showed that the level of MDA, an indicator of lipid peroxidation, were lower in the kidney of the GSPE + Pb group compared with the Pb group. Biochemical and biophysical findings suggest a detailed model of the composition and structure of membranes, which includes levels of dynamic organization both across the lipid bilayer (lipid asymmetry) and in the lateral dimension (lipid domains) of membranes [26]. Lipids play a crucial rule in the structure of cell membranes, suggesting that GSPE may plays a potential protective role in the cell membrane to inhibit the entry of Pb into cells.

MDA is one of the most important biomarkers in oxidative damage [27]. Antioxidant defense systems include enzymatic (SOD and GST) and nonenzymatic (GSH) antioxidant mechanisms [28]. Our results suggested that oxidative stress was involved in the pathophysiology of Pb-induced renal injury. In the present study, increased MDA level, as well as decreased GSH concentration and activities of SOD and GST induced by Pb were consistently relieved by GSPE treatment. Thus, the antioxidant properties of GSPE appeared to underlie the mechanism of protective effects against kidney injury induced by Pb.

A potential mechanism of excessive inflammation due to activation of NF-κB by oxidative stress supports the link between inflammation and oxidative stress in the progression of disease [29]. The transcription factor NF-κB plays a critical role in the activation of down-stream genes responsible for secreting pro-inflammatory cytokines, so that it mediates many pro-inflammatory and cell death pathways [30]. TNF-α, a pro-inflammatory cytokine, which can stimulate fibroblasts, endothelial cells, and macrophages to produce chemokines, resulted in tissue damage and chronic inflammation [31]. Our results showed that Pb exposure resulted in intracellular superoxide production, whereas GSPE efficiently suppressed MDA, which was accompanied by the down regulation of NF-κB and TNF-α. In this study, Pb exerted an effect in renal tissues by oxidative stress, and the subsequent NF-κB activation resulted in inflammatory. Our results demonstrate that GSPE exerts a significant suppressive effect on inflammatory induced by Pb.

Oxidative stress is a known promoter of apoptosis, the release of inflammatory cytokines and mediators are associated with the pathogenesis of Pb induced apoptosis [32]. P53 activation triggers apoptosis through activating pro-apoptotic proteins [33]. Based on our results, P53-dependent cell death was involved in Pb-induced nephrotoxicity, which leading to the activation of Bax. Bax harbors a highly conserved domain, which promotes the progress of apoptosis by directly inducing cytochrome c release from mitochondria or suppressing anti-apoptotic activity by forming Bax-Bcl-2 heterodimers [34]. Our data showed that the modulation of pro-apoptotic and anti-apoptotic protein expression in rat kidney after Pb treatment was reversed by GSPE, revealing the anti-apoptotic effect of this compound in Pb-induced kidney injury. A direct link between Nrf2 and both Bcl-2 and Bcl-xl expression was previously shown, because these two anti-apoptotic proteins are under transcriptional control by Nrf2, which binds to antioxidant response element (ARE) located in the promoter region of the corresponding genes [35]. A possible explanation is that GSPE protects renal cells against apoptosis induced by Pb via activating the Nrf2 pathway.

Nrf2 accumulates and translocates to the nucleus where it heterodimerizes with small Maf or other uncertain nuclear proteins, binds to ARE, and finally activates the downstream genes [36]. In the present study, GSPE increased the content of Nrf2 in nucleus, as well as reversed the Pb-induced decrease of the levels of HO-1 and NQO1, the activity of GST, and the concentration of GSH, which are downstream targets of Nrf2. Thus, GSPE plays a critical role in the activation of Nrf2, which attenuates Pb-induced nephrotoxicity by antioxidant, anti-apoptosis, and detoxification mechanisms. Keap1 is in the off position and functions as an E3 ubiquitin ligase, constantly targeting Nrf2 with Cul3-Rbx1 for ubiquitination and proteasomal degradation [37]. However, in this study, administration of GSPE increased Keap1 level in Pb-treated rat kidney, so GSPE may activate Nrf2 pathway in Keap1-independent manner.

miRNAs are small segments of noncoding RNA that regulate gene expression and protein function, and therefore are key regulators of diseases [38]. Nrf2 pathway was found to be negatively regulated by miRNAs. It was reported that miR153 suppressed Nrf2 gene expression through 3′ untranslated region binding and down-modulating Nrf2 mRNA [39]. In this study, higher expression of miR153 and decreased Nrf2 level have been observed in the kidney of rats treated by Pb, GSPE reversed the increase of miR153 induced by Pb as expected. Thus, GSPE activate Nrf2 pathway at least partially through inhibiting miR153.

Suppression of proteasomal degradation of Nrf2 could activate Nrf2 pathway [40]. GSK-3β phosphorylates Nrf2, leading to exclusion of Nrf2 from the nucleus and promoting Nrf2 ubiquitination [36, 41]. It has been reported that N-terminal region of Nrf2 initiates rapid proteolysis within the cytoplasm, but mediates turnover via a slower pathway in the nucleus [42]. Here, the result showed that Pb stress in rat renal tissues significantly decreased the level of p-GSK-3β. The activity of GSK-3β can be inhibited by phosphorylation. Therefore, GSK-3β mediates the nephrotoxicity of Pb via inhibiting Nrf2 pathway. Surprisingly, GSPE promoted phosphorylation of GSK-3β, indicating it might be an effective inhibitor of GSK-3β. Accordingly, GSPE suppresses proteolytic degradation of Nrf2 induced by Pb via inhibiting the activity of GSK-3β, thereby increasing Nrf2 nuclear translocation.

**Figure 8:**
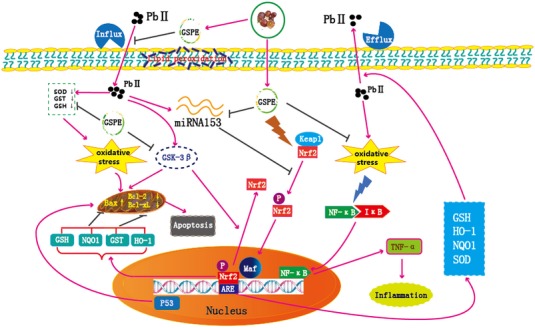
Schematic diagram of the protective mechanism for GSPE in Pb-induced nephrotoxicity *Peachblow line denotes stimulation, brown line denotes inhibition, “
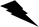
” denotes dissociation*.

In summary, Pb-induced oxidative stress in rat kidneys was attenuated by GSPE treatment via activating the Nrf2 signaling pathway involved in suppression of miR153 and GSK-3β (Figure 8). Nrf2 signaling provides a new therapeutic target for renal injury induced by Pb, and GSPE could be a potential natural agent to protect against Pb-induced nephrotoxicity.

## MATERIALS AND METHODS

### Animals and treatment

All animal procedures were conducted in accordance with the guidelines of the Ethical Committee for Animal Experiments (Northeast Agricultural University, Harbin, China). Healthy male albino Wistar rats (6 to 8 weeks of age, with average body weight of 110 ± 10 g) were used in this study. The animals were acclimated for 7 days under the same laboratory conditions with a 12-h interval light/dark cycle, a minimum of 40 % relative humidity, and a room temperature of 21 ± 4°C. Food (standard diet) and water were available and libitum. The rats were randomly divided into 4 groups of 10 animals each and were treated for 5 weeks as follows: control, Pb-treated, Pb + GSPE, and GSPE. The control group received tap water and was given 0.9 % physiological saline by intragastric administration. The Pb group received an aqueous solution containing 2,500 ppm of Pb acetate (0.25 %, w/v) and was given 0.9 % physiological saline. The Pb + GSPE group was cotreated with Pb (as in Pb group) plus GSPE (200 mg/kg, gastric gavage). The GSPE group received tap water and was given GSPE (200 mg/kg) by intragastric administration. Lead acetate was purchased from the Benchmark Chemical Reagent co., Ltd (Tianjin, China). Grape seed procyanidin was obtained from the Niulan Biological Products co., Ltd (Changsha, China). At the end of the treatment period, the animals were weighed and anesthetized with ether.

### Biochemical analysis

Blood samples were collected from the jugular vein into evacuated tubes that contained heparin sodium as an anticoagulant. The samples were centrifuged at 3,000 rpm for 10 min within 1 h after collection. Serum samples were collected to determine the CREA and BUN levels using UniCel DxC800 Synchron (Bekman, USA). Renal tissues were rapidly excised and weighted then homogenized in phosphate-buffered saline (PH 7.4, w/v; 1 g tissue with 9 ml PBS) with an Ultra-Turrax T25 Homogenizer. After centrifugation at 3,500 rpm for 10 min at 4°C. The supernatant was used to determine the MDA and GSH concentration levels and activities of the antioxidant enzymes (i., SOD, GST), which were measured using assay kits from Nanjing Jiancheng Bioengineering Institute (Nanjing, China) according to the manufacturer's instructions.

### Histopathological analysis

The 10 % neutral-buffered formalin solution-fixed kidneys were embedded in paraffin; sections with a thickness of 5-6 μm were sliced from the paraffin-embedded blocks and stained with hematoxylin and eosin stain. Then, the histological slides were examined by light microscopy (BX-FM; Olympus Corp, Tokyo, Japan).

### Total Pb analysis in the kidney

Briefly, approximately 0.5 g renal tissues were ashed at 500°C for 16  h. Ashed samples were digested by heating to a slow boil in a very small amount of perchloric acid and nitric acid over 4-6 h. 10 ml of hydrochloric acid were added, and water was added to concentrated samples (50 ml). Absorbance readings were performed with a 930 System Atomic Fluorescence Spectrometer.

### TUNEL assay

The detection of apoptosis was performed with a TUNEL detection kit (Kaiji, Nanjing, China). The procedure was conducted according to the manufacturer's instructions. Sections (4 μm) from the paraffin-embedded blocks were dewaxed and added non DNase protease K. Nest sections were rinsed 3 times with PBS, labeled at 37°C for 60 min with the TUNEL reaction mixture, and rinsed again with PBS. The number of apoptotic cell was counted from 10 to 20 random fields (200×) of a coverslip. Cells were examined and recorded under a fluorescence microscopy.

### Western bolt analysis

Total protein of kidney tissues was lysed in RIPA buffer supplement with the protease inhibitor PMSF (Beyotime, Shanghai, China). Briefly, the homogenate was centrifuged at 12,000 rpm for 10 min at 4°C and supernatant was collected. Nuclear and cytosolic proteins were extracted using a Nuclear and Cytoplasmic Protein Extraction Kit (Beyotime) following the manufacturer's instruction. Total protein content was determined using BCA protein assay kit (Beyotime). Equal amounts of sample (10 μl, 3 mg/ml) were loaded on SDS-PAGE and transferred to PVDF membranes. After blockage of nonspecific binding sites with 5 % nonfat milk in TBST (TBS and 20 % Tween 20) for 2 h at room temperature, membranes were incubated overnight at 4°C with diluted Primary antibodies against-Bax, Bcl-2, Bcl-xl, P53, P-GSK-3β (Ser9), GSK-3β, NF-κB, TNF-α, Nrf2, Keap1, NQO1, and HO-1, respectively. Above primary antibodies against each protein were from Santa Cruz Biotechnology (Santa Cruz, CA, USA). Nuclear protein Lamin B (Santa Cruz, CA, USA) and GAPDH (Xianzhi, Hangzhou, China) were used as loading controls. The membranes were then washed 4 times with TBST, incubated further with the appropriate horseradish peroxide conjugated secondary antibody at 37°C for 45 min, and then washed 5 times with TBST. Subsequently, bands quantified using Image Pro-Plus 6.0 software (General Electric Company, Fairfield, CT, USA).

### miR153 isolation and relative quantitative real-time PCR analysis

miR153 was extracted using SanPrep Column miRNA Mini-Preps Kit (Sangon Biotech, Shanghai, China) according to the manufacturer's instructions. For real time PCR analysis of miR153, cDNA was used. miRNA detection by real time analysis involved reverse transcription of cDNA using a small RNA specific stem-loop RT primer (5′-CTCAACTGGTGTCGTGGAGTCGGCAATTCAGTTGAGGATCACT-3′). The thermal cycling included 3 min of denaturation at 95°C followed by 45 PCR cycles, including 15 s at 95°C, 20 s at 57°C, and 30 s at 72°C. The comparative Ct (2^−ΔΔCt^) method was used to analyze the relative expression of miR153, which were normalized to U6.

### Statistical analysis

Statistical analyses were performed using SPSS 19.0 software (SPSS, Chicago, IL, USA). All datas are expressed as mean ± SEM. Statistical analysis was performed by one-way ANOVA (analysis of variance) and using Tukey's post hoc test to assess significance. Values with P < 0.05 were considered as statistically significant.

## Abbreviations

BUN: blood urea nitrogen;
CREA: creatinine;
GSH: glutathione;
GSK-3β: glycogen synthase kinase 3β;
GSPE: grape seed procyanidin extract;
GST: glutathione S-transferase;
HO-1: heme oxygenase-1;
MDA: malondialdehyde;
NF-κB: nuclear factor-κB;
NQO1: NAD(P)H quinone oxidoreductase 1;
Nrf2: nuclear factor-erythroid-2-related factor 2;
Pb: lead;
SOD: superoxide dismutase;
TNF-α: tumor necrosis factor-α
